# A Field Experimental Design of a Strengths-Based Training to Overcome Academic Procrastination: Short- and Long-Term Effect

**DOI:** 10.3389/fpsyg.2017.01949

**Published:** 2017-11-07

**Authors:** Lennart Visser, Judith Schoonenboom, Fred A. J. Korthagen

**Affiliations:** ^1^Graduate Masters Programme for Teachers, Driestar Christian University for Teacher Education, Gouda, Netherlands; ^2^Department of Education, University of Vienna, Vienna, Austria; ^3^Social and Behavioural Sciences, Utrecht University, Utrecht, Netherlands

**Keywords:** academic procrastination, intervention study, positive psychology, core reflection, character strengths

## Abstract

This study reports on the effect of a newly developed 4-week strengths-based training approach to overcome academic procrastination, given to first-year elementary teacher education students (*N* = 54). The training was based on a strengths-based approach, in which elements of the cognitive behavioral approach were also used. The purpose of the training was to promote awareness of the personal strengths of students who experience academic procrastination regularly and to teach them how to use their personal strengths in situations in which they usually tend to procrastinate. With a pretest-posttest control group design (two experimental groups: *n* = 31, control group: *n* = 23), the effect of the training on academic procrastination was studied after 1, 11, and 24 weeks. Results of a one-way analysis of covariance revealed a significant short-term effect of the training. In the long term (after 11 and 24 weeks), the scores for academic procrastination for the intervention groups remained stable, whereas the scores for academic procrastination for the control group decreased to the same level as those of the intervention groups. The findings of this study suggest that a strengths-based approach can be helpful to students at an early stage of their academic studies to initiate their individual process of dealing with academic procrastination. The findings for the long term show the importance of measuring the outcomes of an intervention not only shortly after the intervention but also in the long term. Further research is needed to find out how the short-term effect can be maintained in the long-term.

## Introduction

Each year, many first-year students in higher education are not successful in their courses and (have to) drop out of school. There are many reasons for dropping out. One of the causes of academic failure among students is academic procrastination (Solomon and Rothblum, 1984; Steel, 2007). When students procrastinate, they are passive in starting or completing academic tasks. Academic procrastination can have negative financial and personal consequences for students and financial consequences for institutions. Consequences of academic procrastination for students are under-performance, low grades on tests and final exams (Steel et al., 2001), and an increased risk of dropping out (Wesley, 1994). In a meta-analysis, Steel (2007) cited research estimating that 80–90% of undergraduate college students experience some form of academic procrastination (Ellis and Knaus, 1977; O'Brien, 2002). Research among college students shows that nearly all college students admit to procrastinating at least occasionally and that 42% usually or always procrastinate (Zarick and Stonebraker, 2009). Concurrent with Klingsieck (2013), we define procrastination as the voluntary delay of an intended and necessary and/or (personal) important activity, despite expecting potential negative consequences that outweigh the positive consequences of the delay. In the case of *academic procrastination*, this concerns the delaying of academic study activities.

Various predisposing, causing, and maintaining factors can influence a person's procrastination (Van Eerde, 2003; Steel, 2007; Klingsieck, 2013; Egan et al., 2014; Rozental and Carlbring, 2014; Steel and Klingsieck, 2016). These factors are within the person, and situational or contextual factors.

### Personal factors

Regarding personal factors, much research has shown that personality traits are related to procrastination (Van Eerde, 2003). The important personality traits predicting procrastination are impulsiveness (*r* = 0.41) and (lack of) self-control (*r* = −0.58). These traits have moderate to strong correlations with procrastination (Steel, 2007) and are often viewed as key factors in other behavioral problems related to self-regulation (Moffitt et al., 2011). People who are more impulsive are more likely to procrastinate. People who are more in control of themselves are less likely to procrastinate. Research using the big five taxonomy showed that conscientiousness has the strongest negative relation (*r* = −0.62) with procrastination (Van Eerde, 2003). People who are more conscientious are less likely to procrastinate. Persons who are conscientious are careful, thorough, and tenacious, which limits the tendency to procrastinate (Ozer and Benet-Martínez, 2006). Neuroticism has a weak association with procrastination (*r* = 0.24). People with neuroticism experience feelings of anxiety and depression. They are more self-conscious and worrisome, which could explain the relation to procrastination (Hettema et al., 2006). Weak correlations on the Big Five scale have been found between procrastination and openness to experience (*r* = 0.03), extraversion (*r* = −0.12) and agreeableness (*r* = −0.12; Steel, 2007).

In addition to personality traits, procrastination is influenced by negative beliefs people have about themselves; for example, assumptions and negative thoughts often result in the delay of commitments (Pychyl and Flett, 2012). When procrastinators experience problems in their performance, they are at risk of self-defeating behaviors because they doubt themselves and experience a low level of self-efficacy (Ferrari and Tice, 2000). Believing in one's ability to perform a given course of action, so-called self-efficacy, is important in order to carry out many of the tasks people face. High self-efficacy (*r* = −0.38) and high self-esteem (*r* = −0.27) prevent the postponing of activities because of irrational beliefs (Steel, 2007).

Procrastination can also be caused by a failure in motivation in the person, which leads to an intention–action gap (Steel, 2007). The need for achievement, so-called achievement motivation, is moderately correlated with procrastination (*r* = −0.35; Steel, 2007). People who are intrinsically motivated experience less procrastination (*r* = −0.26).

### Situational factors/contextual factor

Procrastination can also be understood as a phenomenon evoked by situational features (Klingsieck, 2013). These features can be task characteristics, such as task difficulty and attractiveness, plausibility of the assignment, autonomy, and teachers' characteristics (Ackerman and Gross, 2005, 2007). When a person experiences a task as aversive, it can be more attractive to postpone or delay this task than to start it. The unpleasantness of a task, a person's boredom, and lack of interest can be reasons to procrastinate on a task (Ferrari and Scher, 2000).

### Interventions

Many intervention studies targeting academic procrastination have been conducted. The approaches these studies follow and the underlying change models vary, such as a time-management approach (Engberding et al., 2011; Dryden and Sabelus, 2012; Höcker et al., 2012; Häfner et al., 2014), an approach for emotion regulation (Eckert et al., 2016), an approach for recognizing irrational beliefs (Uzun Ozer et al., 2013), acceptance and commitment therapy (ACT; Scent and Boes, 2014; Glick and Orsillo, 2015), mental imagery (Blouin-Hudon and Pychyl, 2017), and strategies for accomplishing goals (Gustavson and Miyake, 2017).

In a meta-analysis of intervention studies targeting procrastination, Van Eerde and Klingsieck (2017) discern five types of interventions: time management, self-management, cognitive behavioral therapy (CBT), paradoxical/coherence/acceptance therapy, and the field of assertiveness/strengths-based approaches and emotion regulation. According to the preliminary results of that study (Van Eerde and Klingsieck, 2017), interventions based on cognitive behavioral therapy (CBT) are the most promising for overcoming procrastination. For example, Uzun Ozer et al. (2013) taught students to identify their personal procrastination patterns and handle their irrational thoughts, using the RET model (Ellis's ABC model; Ellis and Knaus, 1977). Eckert et al. (2016) helped students overcome their procrastination by enhancing emotion regulation skills.

### Study-designs

Studies of procrastination interventions included a broad range of research designs. Some studies (Häfner et al., 2014; Glick and Orsillo, 2015) include a control group, whereas other studies do not (Engberding et al., 2011; Dryden and Sabelus, 2012; Höcker et al., 2012; Uzun Ozer et al., 2013; Scent and Boes, 2014; Gieselmann and Pietrowsky, 2016). Therefore, the possibility that several of the effects reported occurred by chance cannot be ruled out. There are also various differences in the number of participants involved, strategies (individual vs. group-wise, face-to-face vs. online), duration of the interventions, and times of measurement.

As discussed in the previous section, various factors can influence a person's procrastinating behavior simultaneously. In the academic context in which we conducted this study, students may view a task as “too difficult.” However, the difficulty of a task not only depends on the task but is also influenced by how a student estimates his or her personal capability to do the task. Because of individual differences between students, we presume that a “one size fits all approach” would not be successful for overcoming procrastination. A fruitful approach to overcome procrastination should target various perspectives on procrastination, trying tap into essential aspects of procrastination for every individual student (Klingsieck, 2013). This is what we aimed at in our study.

The intervention that we developed and describe in this article is built on principles from positive psychology and combined with elements from CBT. According to the founders of positive psychology (Seligman and Csikszentmihalyi, 2000), “Treatment is not just fixing what is broken; it is nurturing what is best” (p. 9). If the main emphasis in the treatment of persons experiencing a problem is on what is wrong with a person, there can be the risk that possibilities and potential that could be realized through accessing a person's personal strengths receive less attention. Therefore, positive psychology focuses on the mechanisms underlying well-being and personal growth. Positive psychology does not neglect the problems a person experiences but emphasizes the search for possibilities and potential within the person to help the person handle his or her problems. Thus, traditional psychology and positive psychology do not exclude each other but should be seen as complementary (Johnson and Wood, 2017). We believe that integrating both fields of psychology offers new opportunities for the treatment of people who experience problems with procrastination.

One principle that we derive from positive psychology is to build on students' personal strengths, called *character strengths* (Fredrickson, 2009). Character strengths can be defined as positive traits reflected in thoughts, feelings, and behaviors (Park et al., 2004) and are considered an important aspect of people's *psychological capital* (Luthans et al., 2007). Examples of character strengths are curiosity, perseverance, willpower, judgment, wisdom, zest, self-control, enthusiasm, hope, and determination. According to Fredrickson's (2003, 2009) *broaden-and-build theory*, a focus on character strengths and positive emotions expands people's repertoires of thoughts and actions (Fredrickson and Branigan, 2005). This helps people to experience more creativity (Rowe et al., 2007) and become more open to new experiences (Kahn and Isen, 1993).

Other studies show that enhancing people's awareness of their character strengths and promoting them to use these character strengths in a conscious manner have a positive effect on people's optimism (Luthans et al., 2007) and well-being (Seligman et al., 2005). It has also been shown that the deliberate use of character strengths reduces depression (Fredrickson, 2013). Character strengths are also important for developing resilience and are significantly correlated with resilience-related factors, such as self-efficacy, positive affect, self-esteem, and optimism (Martínez-Martí and Ruch, 2017).

With the results of the studies mentioned previously in mind, we hypothesize that when students learn to be aware of their character strengths and use these strengths in situations of academic procrastination, their tendency to procrastinate during academic activities will decrease. Only one intervention study has reported on a strengths-based approach among students experiencing academic procrastination (Ossebaard et al., 2013). The results of this in-depth study (*N* = 6) describing an intervention of 3 weekly meetings 2.5 h long were promising. Each student had lower academic procrastination levels after the intervention. However, the limitations of this study were the small sample size and the lack of a control group.

Because of the promising results of studies on the effect of strengths-based approaches in general, and the limited research on strengths-based approaches to academic procrastination, further research seems important. The present study connects the research field of positive psychology to the research field of academic procrastination. Thus, this study has relevance for both research fields. It has also practical relevance, for example, for staff members of universities, such as teachers or counselors who work with students. To our knowledge, the current study is the first randomized controlled experiment examining the efficacy of a strengths-based approach to overcome academic procrastination in the short and long term.

### Research aim and question

In this study, we examined the effect of a strengths-based training among first-year elementary teacher education students with high levels of academic procrastination. The aim of the study was to explore whether a positive psychological approach would be beneficial to overcome academic procrastination. We wanted see the effect of promoting awareness of character strengths on students' tendency to procrastinate. The central research question was: What is the effect of strengths-based training to overcome academic procrastination on the students' level of academic procrastination?

### Theoretical framework of the intervention

The strengths-based training that we developed aims at reducing students' procrastination behavior. Students are taught to gain insight into how and why they procrastinate and are taught to overcome their procrastination tendency by identifying their character strengths and using them in situations in which the students tend to procrastinate.

Our Strengths-based Training to Overcome academic Procrastination (abbreviated as STOP) is based on the core reflection approach (Korthagen, 2013, 2014), a strengths-based approach in which elements of CBT are incorporated. Important CBT elements within the core reflection approach are that it takes people's thoughts, feelings, and behavior into account and considers these aspects interrelated (Ellis, 1994). To help students get insight into their own thoughts, feelings, and behavior, elements of rational emotive behavioral therapy (REBT; Ellis, 2008) are included in the program. The core reflection approach is also a practical elaboration of elements from positive psychology, in particular the notion of character strengths. Within the core reflection approach, character strengths are termed *core qualities*. Core reflection is aimed at promoting awareness of core qualities and ideals, the latter because ideals create positive feelings and motivation. The assumption is that as a result of inner obstacles, people often use only part of the potential embedded in their core qualities and ideals. By helping people become aware of the tension this creates inside them, psychological growth is enhanced.

When we compare the core reflection approach (Korthagen, 2013, 2014) with the character strengths approach within positive psychology (Seligman, 2002; Seligman et al., 2005), there are also differences. In the character strengths approach, people are supported to be aware of their character strengths and are stimulated to use them in daily life situations (Seligman, 2002; Seligman et al., 2005). In the core reflection approach, there is an additional emphasis on awareness of *ideals*, and on awareness of how *limiting beliefs* narrow the enactment of peoples' core qualities and ideal(s). In order to help people become aware of their core qualities, ideals, and limiting beliefs, it is important that they are aware of their *state of presence* (Scharmer and Senge, 2008). The state of presence beneficial to overcoming academic procrastination is the state of being in which students are present in the here-and-now and aware of their behavior, as well as of choices they can make in the (study) situation. In our training approach to overcome procrastination, we name this state of presence *withitness*. Withitness is a term originally coined by Kounin (1970) to describe a teacher's awareness of what is going on in the classroom.

The principles we used in the intervention for promoting core reflection are based on an extensive discussion of the core reflection approach by Korthagen (2013, p. 39; Meijer et al., 2009, p. 300). Briefly summarized, these principles are as follows: (1) Promote awareness of ideals and core qualities in the person that are related to the situation reflected on. (2) Identify internal obstacles to acting out these ideals and core qualities. (3) Promote awareness of the cognitive, emotional, and motivational aspects embedded in ideals, core qualities, and obstacles. (4) Promote a state of awareness in which the person is fully aware (cognitively and emotionally) of the discrepancy or friction between the person's ideals, core qualities, and the self-created nature of the internal obstacles. (5) Promote trust in the process that takes place from within the person. (6) Support the enactment of the person's one's inner potential within the situation under reflection. (7) Promote autonomy in using core reflection.

**Figure 1:**
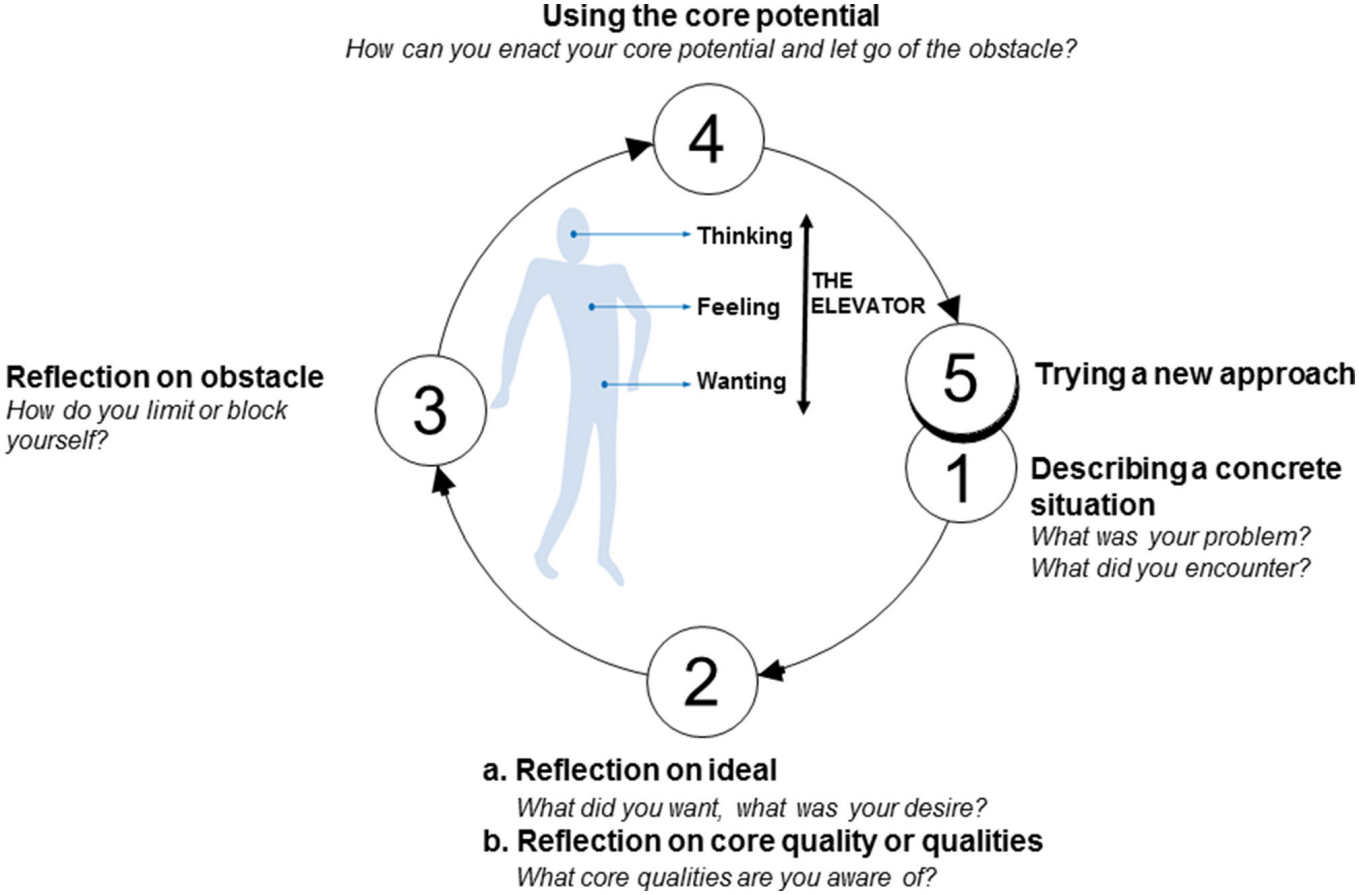
The phase model of core reflection (Korthagen, 2014).

Figure 1 shows the phase model for core reflection (Korthagen, 2014). This figure describes the steps a person goes through during the core reflection process.

Studies on the processes taking place in the core reflection coaching of teachers (Meijer et al., 2009; Hoekstra and Korthagen, 2011) showed that coachees became more mindful of their inner processes and of the relation with their behavior. More specifically, they (1) became more aware of cognitive, emotional, and motivational processes taking place within them, (2) developed an increased focus on their core qualities (e.g., willpower or perseverance) and ideals, and (3) were better able to overcome limiting beliefs and ineffective behavioral patterns. (See Korthagen et al., 2013 for an overview of more studies on core reflection.) We expect that similar processes would occur in the students in the present study. Therefore, we presumed that (1) core reflection would enable students to stay in touch with their ideals and core qualities and (2) deal with limiting cognitive and behavioral patterns, and thus, they would be able to reduce or overcome their tendency to procrastinate. Therefore, our hypothesis was that (a) this effect would occur directly after the STOP training and (b) would still be present after 11 and 24 weeks.

In the Method section, we describe the activities used in the STOP training and we discuss on which of the core reflection principles these activities are based and how the activities are expected to help students deal with or overcome their tendency for academic procrastination.

## Method

### Participants and procedures

The present study took place at a small Christian college for teacher education with approximately 1,500 students, located in the Netherlands. Before we selected the participants for this intervention study, we measured the level of academic procrastination of all first-year students (*N* = 230; 21.2% male; 78.4% female; age between 16 and 27 years, *M* = 17.91, *SD* = 1.24) enrolled in the 4-year elementary teacher education program. One week before the students were asked to complete the questionnaire measuring their procrastination tendency, they were informed by e-mail about the research project and told that their participation was voluntary.

### Sampling method

After we ranked the results of the initial measurement of academic procrastination, we personally asked 67 students with the highest score on academic procrastination if they would be willing to participate in the study. They were informed about the objectives of the study and were given information about who they could contact for questions or comments. Fifty-four students agreed to participate and gave written consent.

To determine the participants for the intervention and control groups, we randomly placed the students in three similar groups, using systematic sampling. Thirty-one students formed two experimental groups: group A (*n* = 14; 4 men, 10 women; age between 17 and 20 years, *M* = 18.07, *SD* = 0.10) and group B (*n* = 17; 4 men, 13 women; age between 17 and 21 years, *M* = 18.18, *SD* = 1.23). A group size of 31 would be too large to be one training group; therefore, two experimental groups were formed. The other students formed the control group (*n* = 23; 1 man, 22 women; age between 16 and 20 years, *M* = 17.78, *SD* = 1.09).

During the academic year in which the research was conducted, several participants dropped out of school and therefore did not participate in the study. In the intervention groups (*n* = 31), seven participants dropped out. Four participants dropped out during the intervention period, and three participants dropped out within 2 months after the intervention. In the control group (*n* = 23), four participants dropped out within 8 months after the intervention period. An independent *t*-test showed that there were no significant differences in scores for procrastination between the students who dropped out of school and the students who did not, neither within the intervention groups and the control group together nor within the intervention groups.

### Debriefing and follow-up care

During the training sessions, all participants in the experimental group were informed about the objectives of the study and about who they could contact with any questions or comments. During the training sessions, there was room for participants to ask questions about the research and the training intervention. After the study was completed, participants were still able to contact one of the trainers in case they had questions or wanted to talk about their procrastination problem. None of the participants used this opportunity to contact the trainer.

### Times of measurement

Students' academic procrastination was measured at four time points during the academic year. At the teacher college where we conducted this study, an academic year is divided into four terms. A term consists of 6 weeks of classes, followed by an examination week and then a 2-week internship. See Table 1 for an overview of the academic year, the measurement points, and the intervention sessions.

**Table 1:** Overview of the academic year, measurement points, and intervention sessions.

**TERM 1**											
Calendar week	36	37	38	39	40	41	42	43	44	45	46
Semester week	1	2	3	4	5	6	Fall holiday	Exams	Internship	Internship	College camp
Research activity								T1 academic procrastination (6 weeks before the intervention)			
**TERM 2**											
Calendar week	47	48	49	50	51	52	01	02	03	04	05
Semester week	1	2	3	4	Christmas holiday	Christmas holiday	5	6	Exams	Internship	Internship
Research activity			STOP Session 1	STOP Session 2			STOP Session 3	STOP Session 4	T2 academic procrastination (1 week after the intervention)		
**TERM 3**											
Calendar week	06	07	08	09	10	11	12	13	14	15	
Semester week	1	2	Holiday	3	4	5	6	Exams	Internship	Internship	
Research activity								T3 academic procrastination (11 weeks after The intervention).			
**TERM 4**											
Calendar week	16	17	18	19	20	21	22	23	24	25	26
Semester week	1	2	Holiday	3	4	Internship	Internship	Internship	5	6	Exams
Research activity											T4 academic procrastination (24 weeks after the intervention).

All the respondents completed the questionnaires on one of the first 2 days of the exam week at the end of periods 1, 2, 3, and 4. When the students had finished the exam, they filled out the questionnaire before they left the examination room. All questionnaires were completed in the examination room, where the students were not allowed to talk to each other and therefore could not influence one another. The role of the monitors present was limited to distributing and collecting the questionnaires.

One week before the students were asked to complete the questionnaire measuring their procrastination tendency, they were reminded by e-mail about the research project. At all measurement points, the respondents were reminded by the written introduction to the questionnaire that participation was voluntary. They were also informed about the purpose of the research, the expected duration and the procedure, confidentiality, and the promise that the data would be processed anonymously. If a student had any questions, he or she could contact the first author of this study by e-mail or phone.

### Instrumentation

In line with previous research (Höcker et al., 2008; Krause and Freund, 2014), the degree of academic procrastination was measured with items of the subscale procrastinatory study behavior of the Dutch Academic Procrastination State Inventory (APSI; Schouwenburg, 1994, 1995). The original reliability coefficient of the 15 items regarding procrastination study behavior was α = 0.91 (Schouwenburg, 1994, 1995). The items asked the respondents about their study behavior during the week before they completed the questionnaire. Each item begins with the question “How often did you…last week?” On a five-point Likert scale ranging from never (1) to always (5), the student indicates his or her assessment of how often something happened. Higher scores indicate a higher tendency to procrastinate. Sample questions include the following: “How often during last week did you not study the material that you had planned to study?” and “How often during last week did you do so many other things that you had too little time left for your studies?”

### Data analysis

Data were analyzed in SPSS 21. After checking the ANCOVA assumptions (Field, 2013) for (1) independence of the covariate and treatment effect, and (2) homogeneity of regression slopes, we used a one-way analysis of covariance (ANCOVA) to determine statistically significant differences between mean test scores of the variable academic procrastination of the experimental and control groups, at T2, T3, and T4, controlling for the test scores at T1. Participation by the experimental groups was used as a fixed factor.

### Structure of the training sessions

Participants in the intervention group followed four 3-h group meetings, spread over a period of 6 weeks. The training sessions were developed and given by the first and third author of this article. The pedagogical approach used in the training session can be defined as learning by doing (Gibbs, 1988). This means that during the sessions theory and practice were used alternately to teach participants the principles of the core reflection approach. The participants were stimulated to practice the principles in study activities in the periods between sessions. During the STOP training, the trainers did not train traditional study skills such as planning, organizing study activities, or managing time. Participants learned to choose their own set of core qualities, matching one or more factors that caused their procrastination. The rationale behind this individualized approach is that the student is an expert regarding his or her own procrastination, and thus, each student choosing his or her own solutions to overcome his or her procrastination behavior is the most helpful approach.

We wanted students to explore what maintained their tendency to procrastinate and which internal and/or external factors play a role. Students learned that they are the expert on their own procrastination problem and that the solution to overcoming their procrastination problem is within them. The idea of the intervention is that students discover the underlying factors of their procrastination behavior by becoming aware of what they do, what they think, and what they want, and learn how to handle these factors. Students thus are engaged in their own treatment and learn to change the perspective on their procrastination problem toward regarding it as manageable, instead of unchangeable.

Each session started with a reflection on a positive study experience during the previous week(s), leading to the question of what core qualities were used in that situation. In every meeting, the core reflection approach was demonstrated by one of the trainers through coaching of a participant. A demonstration was followed by a group reflection on what the participants observed and what principles were important in the demonstration coaching. After this reflection, all participants practiced the approach in pairs. During the sessions, the participants were asked to reflect and write down helpful ideas and reflections in personal logbooks, with a focus on the question of how they could coach themselves during the next week. Each session ended with an individual reflection on what the participants had learned during the particular session and how they wanted to apply this learning in the upcoming week.

#### Sessions

##### Session 1: discovering one's core qualities

The first meeting started with the group-forming process. The trainers and the participants introduced themselves. Participants were informed about the research and about the planning of the four sessions. General group guidelines were introduced, including confidentially, mutual respect, and emotional safety. The importance of distinguishing between thinking, feeling, and wanting was explained (principle 3). The notion that people can have various *selves* (Dunkel and Anthis, 2001; Stone and Stone, 2011) was explained and introduced. Participants learned that they have two selves, the *academic procrastination self* and the *control self*. The trainers showed how awareness of thinking, feeling, and wanting could be used to switch from a modus in which the academic procrastination self is in charge to a modus in which the control self takes the lead (principles 1–4).

One trainer demonstrated the principles of the core reflection approach by coaching a student with an actual problematic study experience. The participants reflected on this demonstration in pairs, discussing the following questions: What touched you in this demonstration? What do you recognize in yourself? What can you apply to yourself from this demonstration? After the demonstration, the students practiced the core reflection approach in pairs, supported by the trainers, to become familiar with the approach. Next, a reflection and discussion in the whole group took place, with a focus on how to use the principles that had emerged. At the end of the session, the trainers explained how the logbooks could be used during the time between the sessions to support awareness of the principles the students had learned.

##### Session 2: withitness and taking control

The concept of the two *selves* (the academic procrastination self and the control self) was further elaborated in a role play demonstrated by one of the trainers (principles 1–4). After the demonstration, participants reflected on what they had seen in the role play, based on the leading question: What happened with this person, and what could help this person to get into the study mode? To promote awareness of their level of withitness in the here-and-now and during future moments in which they usually experience the tendency to procrastinate, participants were asked several times (in this session and the following sessions) to score their level of withitness at that particular moment on a scale of 1–10 and show their score to the group with the help of a sheet. The trainers focused on the importance of being aware of one's core qualities during study activities and the crucial role of *feeling* these qualities in the here-and-now. After one of the trainers again demonstrated the core reflection approach, participants practiced the approach in pairs and reflected on their experiences within the whole group (principles 1–7). At the end of the meeting, a guided reflection was used in which the students reflected on a future study moment and on how they could be with it in that moment and what outcome that would yield (principles 1, 3, 4, 6, and 7).

##### Session 3: the power of ideals, and the difference between limiting and helping thoughts

The concept of withitness was refreshed. Participants had to recognize moments of withitness and had to connect them to specific individual core qualities promoting withitness (e.g., the core qualities attentiveness, focus, and awareness) and promoting working under the direction of the control self (e.g., the core qualities goal-directedness, decisiveness, and clarity). Participants learned that moments of withitness and taking control are not just lucky moments but that these moments can be deliberately created by the control self. After one of the trainers had given another demonstration of the core reflection approach, participants again practiced the approach in pairs and reflected on it in the group (principles 1–7). With a guided reflection, participants reflected on their ideals linked to the choice of their study and how awareness of these ideals could be helpful during study activities (principles 1 and 2). Participants discovered which helping thoughts they have in successful study situations and which limiting thoughts played a role in academic procrastination situations. Next, the focus was on how to handle limiting thoughts.

##### Session 4: conversation between the academic procrastination self and the control self

Participants reflected on study situations of the previous week and clarified which helping thoughts they had when their control self was in charge or which limiting thoughts they had when they their academic procrastination self was in charge (principles 3 and 4). Displayed by two chairs representing the control self and the academic procrastination self, participants had to sit on one chair and start a discussion with the other self, represented by the empty chair (principles 1–7). With this exercise, we wanted participants to recognize and further develop their awareness, withitness, and behavior. As the final exercise, the participants were asked to write down a stimulating quote on a “tile” that could help them to take control in problematic study situations.

### Treatment of the control group

The control group was a waiting list control group. They did not receive any treatment. When participants of the control group were invited to participate in the study, they were informed about the possibility to follow the STOP training after the research project was finished.

## Results

### Descriptives

All respondents participating in this intervention study completed the questionnaires at T1 (*N* = 54), T2 (*N* = 46), T3 (*N* = 43), and T4 (*N* = 37). Of all the questionnaires, there were only two instances in which a participant missed one of the 15 items (at T3). The missing scores were replaced through linear interpolation. The reliabilities of the questionnaire (standardized Cronbach's alphas) were excellent (T1: α = 0.90, T2: α = 0.92, T3: α = 0.93, T4: α = 0.92).

### Baseline results

There were no statistically significant differences in academic procrastination in the baseline measurement (T1) between the intervention groups and the control group (*t* = 1.27, *p* = 0.21; Table 2).

**Table 2:** Descriptive data of academic procrastination scores at T1.

**Group**	***n***	***M***	***SD***	***Std. Er*.**	***Min*.**	***Max*.**
Intervention groups	31	48.23	6.52	1.17	37	63
Control group	23	45.96	6.51	1.36	35	61
Total	54	47.26	6.55	0.89	35	63

### Descriptive results at T2, T3, and T4

**Figure 2:**
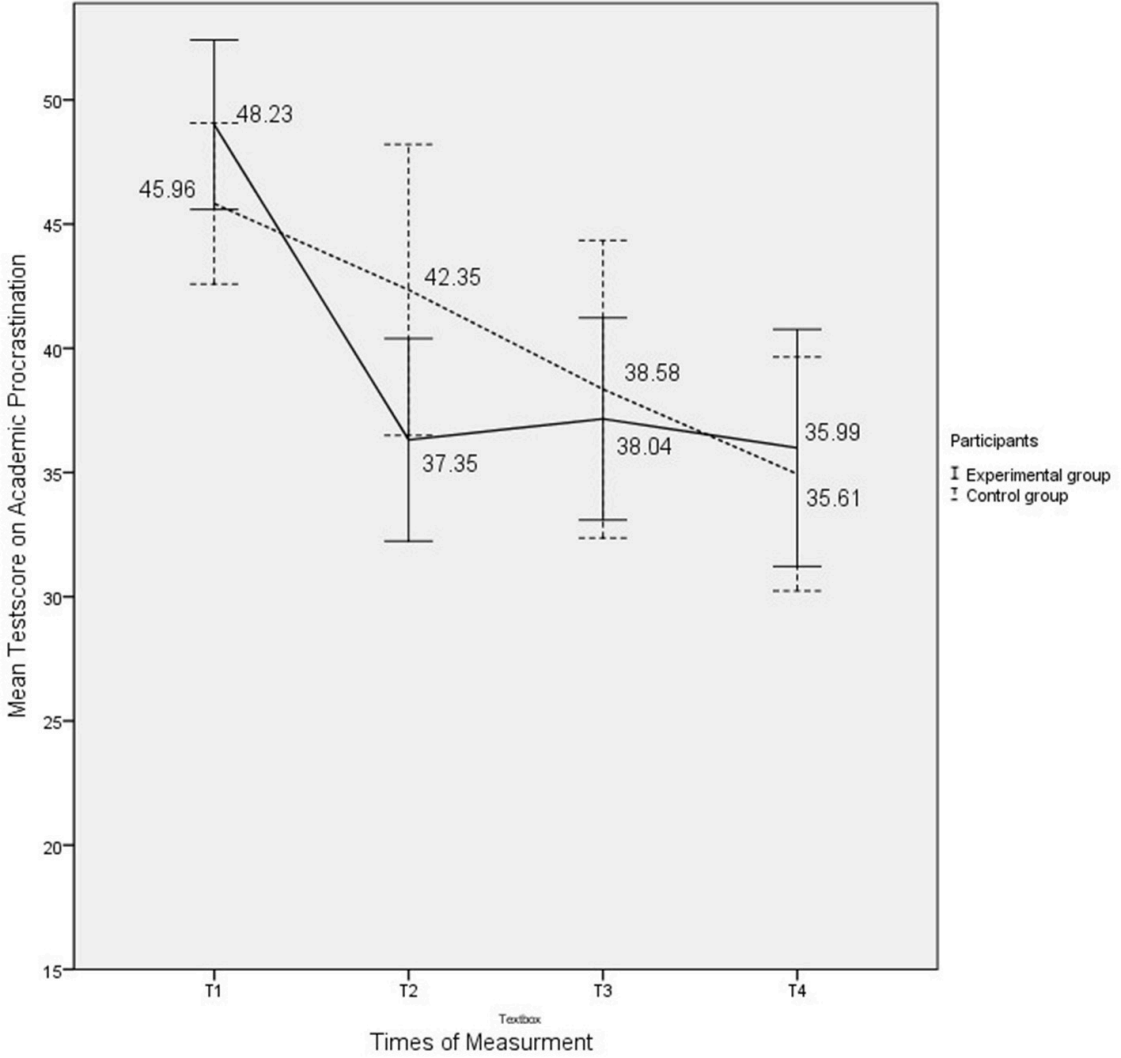
Mean scores before the intervention (T1) and at 1 (T2), 11 (T3), and 24 weeks (T4) after the intervention.

In the experimental group and the control group, procrastination decreased over time. In the experimental group, during the short term a sharp decrease in academic procrastination was observed immediately after the intervention (from 48.23 six weeks before the intervention (*SD* = 6.52) to 37.35 (*SD* = 8.94) 1 week after the intervention had ended). In the control group, the decrease was more gradual from 45.96 six weeks before the intervention (*SD* = 6.51) to 42.35 one week after the intervention (*SD* = 11.83). In the long term, the results of the experimental group showed a slight increase 11 weeks after the intervention (38.04, *SD* = 9.78) and a decrease 24 weeks after the intervention (35.99, *SD* = 10.40). In the long term, the results of the control group, showed a sharp decrease. Measured at T3, i.e., 11 weeks after the intervention, the scores of the control group (38.58, *SD* = 12.08) were almost the same as for the intervention group (38.04, *SD* = 9.78). The scores of the control group at T4, i.e., 24 weeks after the intervention (35.61, *SD* = 9.83) were even lower than those of the intervention group (35.99, *SD* = 10.40) at T4, although not significantly. (See Table 3 and Figure 2.) See Table 4 for correlation coefficients of the intervention group. See Table 5 for correlation coefficients of the experimental group.

**Table 3:** Descriptive results.

**Group**	**T1**	**T2**	**T3**	**T4**
Experimental group	48.23 (6.52), n = 31	37.35 (8.94), *n* = 26	38.04 (9.78), *n* = 24	35.99 (10.40), *n* = 19
Control group	45.96 (6.51), *n* = 23	42.35 (11.83), *n* = 20	38.58 (12.08), *n* = 19	35.61 (9.83), *n* = 18

*Mean Scores and SD (between brackets) Relate to T1, T2, T3, and T4*.

**Table 4:** Correlations of procrastination scores over time: intervention group.

	**T1**	***n***	**T2**	***n***	**T3**	***n***
T1	–					
T2	0.35	26	–			
T3	0.53^**^	24	0.53^**^	24	–	
T4	0.51^*^	19	0.50^*^	19	0.73^**^	19

* *p < 0.05*,

** *p < 0.01*.

**Table 5:** Correlations of procrastination scores over time: control group.

	**T1**	***n***	**T2**	***n***	**T3**	***n***
T1	–					
T2	0.65^**^	20	–			
T3	0.53^*^	19	0.62^**^	18	–	
T4	0.54^*^	18	0.33	17	0.07	18

* *p < 0.05*,

** *p < 0.01*.

### Intervention effects

The results show a statistically significant difference at T2 in mean academic procrastination [*F*_(1, 43)_ = 6.789, *p* = 0.01] between the intervention groups and the control group. The mean academic procrastination decreased by more than seven points (*B* = −7.22, *p* = 0.01). Belonging to an intervention group accounted for 14% of the variance (effect size: eta^2^ = 0.14). At T3 and T4, there was no statistically significant difference between the intervention groups and the control group [T3: *F*_(1, 40)_ = 0.901, *p* = 0.35, and T4: *F*_(1, 34)_ = 0.443, *p* = 0.51; see Table 6].

**Table 6:** Effects of the Intervention at T2, T3, and T4.

**ANCOVA**						**Parameter estimates**							
**Source/Parameter**	**Type III sum of squares**	***df***	**Mean Square**	***F***	***p***	**β**	***B***	**Std. Er**.	***t***	***p***	**Partial eta squared**	**95% confidence interval**	
**Intervention at**												**Lower bound**	**Upper bound**
T2	561.17	1	561.17	6.79	0.01	−0.34	−7.22	2.77	−2.61	0.01	0.14	−12.81	−1.63
T3	79.39	1	79.39	0.90	0.35	−0.13	−2.79	2.94	−0.95	0.35	0.02	−8.74	3.15
T4	34.07	1	34.07	0.44	0.51	−0.10	−1.97	2.96	−0.67	0.51	0.01	−7.98	4.05

## Discussion

In this study, we examined the effect of a strengths-based training approach to overcome academic procrastination on students' level of academic procrastination. The significant differences in short-term academic procrastination scores between the experimental group and the control group show that the STOP training can be effective in diminishing students' academic procrastination. The promotion of awareness of the tension between core qualities and ideals, and inner obstacles, in particular limiting thoughts, in combination with guidelines for overcoming the tension by being aware of one's ideals and character strengths is characteristic of the core reflection approach and appears to have a strong potential for diminishing academic procrastination behavior. These results make clear that a positive psychological approach focusing on strengths can be beneficial for diminishing students' academic procrastination. In particular, supporting and regenerating character strengths can be an effective approach for overcoming academic procrastination.

The results after 11 and 24 weeks showed that the procrastination score of the control group decreased over time. Therefore, in the short term the training was successful. However, in the long term (11 and 24 weeks after the intervention), the mean test scores of the experimental group and the control group no longer showed a significant difference. In this period at the same time, we see a decrease in the procrastination scores of the students in the control group. The students in the control group seemed to improve their academic procrastination tendency to the same degree as the students participating in the experimental group. It is difficult to explain this finding. A possible explanation is that the intervention groups and the control group learned how to take more control over their own behavior or gradually discovered how important it was to not procrastinate, and that the students in the intervention groups were supported by the intervention and therefore managed to handle their academic procrastination at an earlier stage. Future research could show whether offering follow-up sessions to students in the intervention group further expands the initial effect. Another possible explanation could be that procrastination changes over time. When the exam period is coming closer, students may procrastinate less, because there is a stronger need to come into action. In the present study procrastination was measured in the weeks when students had their exams. Future research could show whether long-term effects of the STOP training differ between the intervention group and the control group when procrastination is measured at other moments, for example in the middle of the term, i.e., a few weeks before the exam period.

Many previous intervention studies (Dryden and Sabelus, 2012; Glick and Orsillo, 2015; Gieselmann and Pietrowsky, 2016) targeting academic procrastination show positive results in the short term. The results of the present study also show effects of the intervention in the long term and make clear that it is important to measure effects procrastination interventions in the long term. In two recent studies (Höcker et al., 2012; Uzun Ozer et al., 2013), long-term effects were measured, but these studies did not include a control group. The results of the present study show that in order to conclude that an intervention is effective, it is necessary to measure outcomes in the long term and to compare the results with those of a control group.

To our knowledge, the current study is the first randomized controlled trial among first-year students that examined the short-, mid-, and long-term effect of the core reflection approach on academic procrastination. The results suggest that this approach can be beneficial. Additional intervention studies should be conducted to learn more about effective ways of dealing with academic procrastination based on the strength-based approach of positive psychology. In particular, emphasizing positive situations instead of negative situations, students' core qualities instead of their weaknesses, and their ideals instead of failures in the past can be a helpful way to change students' thinking, and thus, their academic behavior.

The results of the present study should be interpreted against the background of several limitations. In this exploratory study, we focused on the effect of the training program and did not focus on the process that caused the effect. In future research, the process could be monitored, for example, by measuring whether and how students' withitness improves and what core qualities play a crucial role in overcoming academic procrastination. It would be valuable to identify the key active components of this strength-based approach in more detail. In addition, the reported results are based on the specific context of a small sample of predominantly female participants at a small teacher education college in the Netherlands. The conclusions are related to this specific context and do not allow firm generalizations. The assessment of academic procrastination by using the APSI questionnaire may also be limited because it is based on self-reports. Participants need to be aware of their behavior and be honest in their reporting to yield accurate responses. In addition, certain demand effects on the outcomes cannot be ruled out.

In this study, we measured academic procrastination during the exam period. During this period, there is high pressure on students to prepare for their exams. Students can believe that working under pressure gives them a last-minute rush. It is possible that students' academic procrastination differs among situations and periods of an academic year. Perhaps the outcomes of the procrastination scores of the students in the intervention groups and the control group would be different if academic procrastination were measured during a week with no pressure to study or to perform study activities. Future research measuring academic procrastination several times during the time span of a year can yield more insight into fluctuations in academic procrastination behavior, as well as into factors that can or cannot be influenced.

Building on the results of the present study, further research is needed to find out whether and if so, how the short-term effect can be maintained in the long-term.

Future research is also needed to determine whether strength-based interventions are more effective than other approaches, for example, approaches targeting emotion and emotion regulation (such as cognitive restructuring) or interventions aiming at the improvement of time management. An important question is also whether various beneficial approaches share a common mechanism that helps students overcome their academic procrastination tendency.

Answering the question of what components of the training did or did not contribute to overcoming students' procrastination is difficult. We obtained the first idea of an underlying mechanism from student reactions during the meetings to our question about what they perceived as the powerful ingredients of the training program. Many reactions showed that being aware of one's core qualities was considered helpful in overcoming academic procrastination. As one student put it: “Really, being aware of my core qualities! I know this now, so I can use that when I am studying. Then I just start studying and know that when I start, I will do it well. That often helps, that helps very often!” Many students told us that before the STOP training, when they were in a tough study situation, they saw only all the work to be done and were not connected with their ideals. Learning to be aware of their ideals helped many students to become more focused and helped them to persevere. Student: “It helps me to see my ideals. Because if you think something like… this is what I want, then you are very aware of it. I just want this and just will do it! That helps to do your assignments with awareness.” Our overall impression from the students' reactions is that the strengths-based approach helps students to see what they *can* do, instead of seeing what they *cannot* do. They are no longer slaves to their procrastination tendency but are aware of their character strengths and the options these character strengths give them to take control over their procrastination behavior. Although these final reflections on our intervention are no more than first impressions of a potential underlying mechanism, they seem sufficiently interesting for further research.

## Ethics statement

This study was carried out in accordance with the recommendations of the local guidelines of the Faculty of Behavioral and Movement Sciences, VU University Amsterdam, version: Nov. 15 2016, which are based on the Code of ethics for research in the social and behavioral sciences involving human participants, as accepted by the Deans of Social Sciences in the Netherlands, January 2016. All subjects gave written informed consent in accordance with the Declaration of Helsinki.

## Author contributions

LV: PhD-candidate at VU University. He is the principal investigator and principal author of this article. He collected the data, co-developed the intervention program, and co-trained the participants in the intervention groups. JS: supervisor and co-writer of the method-, results/data analysis- and discussion section. FK: supervisor and co-writer of parts of the manuscript. He co-developed the intervention program, and co-trained the participants in the intervention groups.

### Conflict of interest statement

The authors declare that the research was conducted in the absence of any commercial or financial relationships that could be construed as a potential conflict of interest.
